# Hospital Meetings

**Published:** 1906-04-28

**Authors:** 


### HOSPITAL MEETINGS.

### THE GORDON HOSPITAL.

The annual meeting of governors was held at this hospital on April 19. Mr. A. H. Hughes, Chairman of the General Committee, presided. The adoption of the report was moved by the Chairman, who remarked that, although the wards had been closed for a fortnight last spring for repairs and cleaning, there had been practically the same number of in- and out-patients treated at the hospital in 1905 as in 1904. Upwards of 330 in-patients had been admitted, almost all of whom had been operated upon; while upwards of 1,000 persons had been dealt with in the out-patients' department and supplied with necessary medicine, etc. There had been no diminution in the demands made on the institution, and indeed there were always some patients waiting in pain and suffering for a vacant bed. Everyone must admit that the institution was meeting a real want. With regard to finance, they had spent a little more than usual last year, owing to the fact that the building, for lack of funds, had got into a bad state of repair. Their ordinary expenditure was £2,083, and towards this they received from the public in donations and subscriptions only £304. It was lamentable that only one-sixth of what they spent should be contributed by the public, and he considered it a disgrace to this wealthy city. Were it not for the fact that the bulk of the patients contributed towards their board and lodging the work cculd not be carried on for a week. But even with the patients' contributions they could not make both ends meet, and were dependent on the precarious profits of entertainments, etc., got up by their friends. He earnestly implored everyone who took an interest in the hospital to bring the facts of the case before their friends. They were also overburdened with debt, as the building was mortgaged for nearly £6,000. This debt was a source of constant anxiety to the committee. The question of the debt was so urgent that the committee had started this year a special mortgage redemption fund in order to reduce it, and he urged all present to canvass for subscriptions towards this fund. There was a great deal of talk now about efficiency in hospital management, and he was glad to call attention to the fact that they had made economies under the head of housekeeping; these had not been made at the expense of efficiency. The requirements of the medical staff increased yearly, but this could not be prevented, nor would the committee wish to do so, as they felt that the sufferers in the hospital were undoubtedly benefited by every advance in medical science.

Mr. L. N. Earle having seconded the motion, it was carried, as were various other resolutions and votes of thanks.

